# Effectiveness of guideline dissemination and implementation strategies on health care professionals’ behaviour and patient outcomes in the cancer care context: a systematic review protocol

**DOI:** 10.1186/s13643-015-0100-9

**Published:** 2015-08-25

**Authors:** Jennifer R. Tomasone, Rushil Chaudhary, Melissa C. Brouwers

**Affiliations:** School of Kinesiology & Health Studies, Queen’s University, Kingston, ON K7L3N6 Canada

**Keywords:** Clinical practice guidelines, Cancer care, Dissemination interventions, Implementation interventions, Medical health care professionals, Allied health care professionals

## Abstract

**Background:**

Health care professionals (HCPs) are able to make effective decisions regarding patient care through the use of systematically developed clinical practice guidelines (CPGs). These recommendations are especially important in a cancer health care context as patients are exposed to a multitude of interdisciplinary HCPs offering high-quality care throughout diagnosis, treatment, survivorship and palliative care. Although a large number of CPGs targeted towards cancer are widely disseminated, it is unknown whether implementation strategies targeting the use of these guidelines are effective in effecting HCP behaviour and patient outcomes in the cancer care context. The purpose of this systematic review will be to determine the effectiveness of different CPG dissemination and implementation interventions on HCPs’ behaviour and patient outcomes in the cancer health care context.

**Methods/design:**

Five electronic databases (CINAHL, the Cochrane Controlled Trials Register, MEDLINE via Ovid, EMBASE via Ovid and PsycINFO via Ovid) will be searched to include all studies examining the dissemination and/or implementation of CPGs in a cancer care setting targeting all HCPs. CPG implementation strategies will be included if the CPGs were systematically developed (e.g. literature review/evidence-informed, expert panel, evidence appraisal). The studies will be limited to randomized controlled trials, controlled clinical trials and quasi-experimental (interrupted time series, controlled before-and-after designs) studies. Two independent reviewers will assess articles for eligibility, data extraction and quality appraisal.

**Discussion:**

The aim of this review is to inform cancer care health care professionals and policymakers about evidence-based implementation strategies that will allow for effective use of CPGs.

**Systematic review registration:**

PROSPERO CRD42015019331

**Electronic supplementary material:**

The online version of this article (doi:10.1186/s13643-015-0100-9) contains supplementary material, which is available to authorized users.

## Background

Clinical practice guidelines (CPGs)—‘systematically developed statements to assist practitioner and patient decisions about appropriate health care for specific clinical circumstances’ [1]—provide health care professionals (HCPs) with synthesized, evidence-based guidance for making decisions regarding the care of patients. The use of CPGs has the potential to improve quality of health care delivery and patient outcomes [2]. The development of a CPG does not, however, guarantee its use in practice without targeted dissemination and implementation interventions [3], or purposeful strategies for achieving practice adherence with a guideline recommendation. Over the past few decades, a burgeoning interest in the effectiveness of CPG dissemination and implementation strategies has led to systematic reviews examining the impact of such interventions on medical (e.g. [4]) and allied HCPs (e.g. [5]), as well as to reviews of reviews (e.g. [6]). However, a review examining the effectiveness of CPG dissemination and implementation interventions specifically targeting HCPs in the cancer care context has yet to be conducted.

The cancer health care context is unique as a number of medical and allied HCPs are involved in different stages of the cancer care continuum (from patients’ diagnosis, treatment, survivorship, to palliative care), and have to function as a multidisciplinary team to provide optimal care [7, 8]. Further, as advances in cancer treatment, and thus the evidence base underpinning CPGs, continue to evolve at a rapid pace, CPGs require routine and frequent updating to ensure recommendations remain current and valid [9, 10]. As a consequence, this presents unique challenges for adopters of recommendations, requiring for instance, HCPs in this context to stay abreast of the most recent best-practice recommendations and be nimble enough to accommodate refinements or changes to their practice routines. An understanding of the effectiveness of CPG guideline and dissemination strategies specifically within the cancer care context can inform the development of such interventions in the future. Therefore, the purpose of this systematic review will be to determine the effectiveness of different CPG dissemination and implementation interventions on HCPs’ behaviour and patient outcomes in the cancer health care context.

## Methods/design

This systematic review is informed by reviews that synthesized literature examining the effectiveness of guideline dissemination and implementation interventions on medical [4] and allied HCPs [5] across all health care contexts. To ensure methodological rigour, the results of this review will be reported according to the Preferred Reporting Items for Systematic Reviews and Meta-Analyses (PRISMA) guideline [11]. This systematic review protocol is registered in the PROSPERO database (CRD42015019331).

### Eligibility criteria

Studies will be selected according to the criteria outlined below.

#### Study designs

Peer-reviewed and published experimental (randomized controlled trials, controlled clinical trials) and quasi-experimental (interrupted time series, controlled before-and-after designs) studies will be included in the search. We will exclude cross-sectional, cohort, qualitative only, retrospective, and case studies, as well as any studies without primary data (e.g. editorials, commentaries). Unpublished data, abstracts and conference proceedings will also be excluded.

#### Participants

Studies examining the dissemination and/or implementation of CPGs among medically qualified (e.g. physicians, nurses) and allied HCPs (e.g. dietetics, rehabilitation therapy, pharmacy, psychology, radiography, social work) who work within the cancer care context will be included. Studies evaluating the introduction of CPGs targeting patients, the general public, or hospital administrators or policymakers (who are not HCPs themselves), without targeting HCPs, will be excluded.

#### Interventions

Guidelines, clinical guidelines, practice guidelines, guidance, advice, recommendations, expert opinion and consensus statements will be considered under the ‘CPG’ umbrella if the study reports that they were systematically developed (e.g. literature review/evidence-informed, expert panel, evidence appraisal). All CPG dissemination and implementation interventions/strategies will be included and classified according to the Mazza taxonomy [12]. The Mazza taxonomy builds upon the Cochrane Effective Practice and Organization of Care (EPOC) group’s data collection checklist [13], and categorizes 49 types of CPG dissemination and implementation strategies targeting HCPs into professional, organizational, financial, and regulatory approaches. Mazza’s [12] taxonomy has been peer-reviewed and pilot-tested [14], and has been used to classify CPG implementation interventions in other systematic reviews (e.g. [15]). Interventions that are not designed to enhance the dissemination and/or implementation of a CPG will be excluded.

#### Comparators

Within each study, separate comparisons will be made for the following: (1) single intervention vs. no-intervention control; (2) single intervention vs. control that receives some intervention; (3) multifaceted intervention vs. no-intervention control; and (4) multifaceted intervention vs. control that receives some intervention.

#### Outcomes

The primary outcome will be an objective measure of HCP behaviour in line with the CPG recommendation (e.g. diagnosis, test ordering, referrals, procedures, prescribing, general management of cancer/chronic disease, patient education/advice). Secondary outcomes will include patient outcomes (e.g., quality of life, less recurrence of cancer) resulting from dissemination and use of the CPG by HCPs.

#### Timing

There will be no restrictions by length of follow-up of outcomes; however, maintenance of outcomes will be noted in final manuscript.

#### Setting

To be included in the review, the dissemination and/or implementation strategies must have been implemented in any cancer setting or context related to cancer care.

### Search strategy

We will search MEDLINE (OVID interface), EMBASE (OVID interface), PsycINFO (OVID interface), CINAHL, and the Cochrane Controlled Trials Register. The literature search will be limited to the English language (given resources are not available for translation) and human subjects. To exclusively capture studies that were not previously included in last systematic review of the effectiveness of dissemination and implementation strategies for CPGs among medical HCPs [4], the literature search will be limited to articles published since 1998.

A search strategy will be built based on the EPOC Group strategy [16] and previous systematic reviews examining guideline dissemination and implementation among medical and allied HCPs ([4] and [5], respectively). The search strategy will combine terms relevant to guideline implementation strategies, medical and allied HCPs, outcomes, trial design, and cancer care. The MEDLINE strategy will be developed and then be peer-reviewed by a health sciences librarian with expertise in systematic review searching who is not on our study team. A draft MEDLINE search strategy is included in Additional file 1. Once the MEDLINE strategy is finalized, it will be adapted for the syntax and subject headings of the other databases. To ensure literature saturation, the reference lists of relevant reviews identified through the search will be hand-searched.

### Data extraction

Literature search results will be uploaded to EndNote X7 reference management software. References will be de-duplicated by comparing citation details, such as author names, year of publication, article title, and journal. Two reviewers will independently screen titles and abstracts of the de-duplicated bibliographic records. Full texts of records passing the title and abstract screening level will be retrieved and examined independently by the two reviewers according to the eligibility criteria above. Disagreements at both screening levels will be resolved through discussion. A PRISMA flow chart will outline the study selection process and reasons for exclusions.

A data extraction form will be created based on the data collection checklist used for Grimshaw and colleagues’ [4] review; however, modifications will be made to account for the inclusion of allied HCP and the cancer care context. The form will be pilot tested by two reviewers (JRT, RC) prior to full data extraction. Each reviewer will extract data from the same five full-text articles to determine the feasibility and acceptability of the created data form, and required modifications to the form will be made. The following data will be extracted from each full-text article: (1) study design; (2) quality criteria (based on the Methodological Criteria in the Cochrane EPOC Data Collection Checklist, more information below; [13]); (3) characteristics of participating providers; (4) characteristics of participating patients; (5) setting; (6) intervention characteristics (including characteristics of the CPGs, type of intervention, and comparator group); (7) outcomes (including both HCP outcomes/process measures and patient outcomes, as well as post-intervention follow-up); and (8) results. These variables will be extracted for all studies by one reviewer (RC), and then verified by a second reviewer (JRT) to reduce reviewer errors and bias. Disagreements will be resolved by discussion. Multiple publications of the same study will be identified and noted in the PRISMA flow chart.

### Quality assessment

The Cochrane Collaboration tool for assessing the risk of bias [17] will be used to assess the risk of bias for RCTs. Information about the article’s sequence generation, allocation concealment, blinding, incomplete outcome data (e.g. dropouts and withdrawals) and selective outcome reporting will be extracted, and a judgment will be made as whether the study is at ‘high risk’ or ‘low risk’ of bias. If there is insufficient detail reported, the risk of bias will be noted as ‘unclear’.

The Cochrane Risk of Bias Assessment Tool for Non-Randomized Studies of Interventions [18] will be used to assess the risk of bias for studies using quasi-experimental designs. Information about confounding, selection of participants, measurement of interventions, departures from intended interventions, missing data, measurement of outcomes, and selection of the reported result will be extracted, and a judgment will be made as whether the study is at ‘critical’, ‘serious’, ‘moderate’, or ‘low’ risk of bias for each category. If there is insufficient detail reported for a given category, the risk of bias will be noted as ‘no information’ on which to base a judgment of risk of bias.

Risk of bias will be determined for all studies by one reviewer (RC), and then verified by a second reviewer (JRT). Disagreements will be resolved by discussion.

### Data synthesis

Due to the anticipated heterogeneity in study outcomes and comparator (c.f. [4]) across a small number of existing trials, a meta-analysis will not be conducted [19]. Instead, a systematic description and narrative synthesis will be provided. Tables will be used to qualitatively describe the studies pertaining to study design (experimental or quasi-experimental), population (HCP and patient characteristics), intervention characteristics, comparator, and outcomes (HCPs/process outcomes, and/or patient outcomes). In-text results will first be presented according to type of implementation strategy (e.g. professional vs. financial interventions), then by single vs. multifaceted strategies (with results stratified by ‘no intervention’ and ‘some intervention’ comparators), then by process vs. patient outcomes. If the necessary data are available, subgroup analyses will be done for medically qualified HCPs and allied HCPs, separately.^1^ All studies will be reported, regardless of level of risk of bias; however, we will note where high risk of bias may influence the interpretation of the review findings.

## Discussion

A comprehensive review of the effectiveness of CPG dissemination and implementation strategies in the cancer care context does not yet exist in the literature. Previous reviews examining the effectiveness of CPG implementation interventions have either examined medical HCPs [4] or allied HCPs [5] across all health care contexts. Given the numerous CPGs that are routinely produced and updated to inform decisions in the care of people with cancer and that patients with cancer undergo several transitions in care between a multidisciplinary team of HCPs [7, 8], this review will provide insight into the effectiveness of CPG implementation strategies specific to this context. The findings of this systematic review will have the potential to inform researchers and practitioners who are interested in developing interventions that enhance CPG dissemination and implementation among both medical and allied HCPs’ in the cancer care context.

## Additional file

Additional file 1:
**Medline search strategy.** Search terms and order for searching Medline database. (pdf 147 KB)

## Abbreviations

CPG: clinical practice guideline
EPOC: effective practice and organization of care
HCP: health care professionals
KT: knowledge translation
PRISMA: Preferred Reporting Items for Systematic Reviews and Meta-Analyses

## Footnotes

1 In order to be included in subgroup analyses, studies will have to report outcomes separately (medical vs. allied HCPs). If the study sample is >50 %, medical HCPs and the results are not presented separately for the two types of HCPs, the sample will be grouped as medical HCPs (and vice versa).
