# Supplementary material for: Effectiveness of guideline dissemination and implementation strategies on health care professionals’ behaviour and patient outcomes in the cancer care context: a systematic review protocol
Source: Syst Rev. 2015 Aug 25;4:113. doi: 10.1186/s13643-015-0100-9 (PMC4547433; doi:10.1186/s13643-015-0100-9)
Supplement: Additional file 1: — Medline search strategy. Search terms and order for searching Medline database. (pdf 147 KB) [file 13643_2015_100_MOESM1_ESM.pdf]

ADDITIONAL FILE 1: MEDLINE SEARCH STRATEGY

Effectiveness of guideline dissemination and implementation strategies on health care professionals' behaviour and patient outcomes in the cancer care context

Tomasone, Chaudhary, & Brouwers

1	randomized controlled trials/
2	randomized controlled trial.pt.
3	controlled clinical trial.pt.
4	intervention studies/
5	experiment$.tw.
6	(time adj series).tw.
7	(pre test or pretest or (posttest or post test)).tw.
8	random allocation/
9	impact.tw.
10	intervention?.tw.
11	chang$.tw.
12	evaluation studies/
13	evaluat$.tw.
14	effect?.tw.
15	comparative studies/
16	or/1-15
17	Neoplasms/
18	Adenocarcinoma/
19	Carcinoma/
20	(adenocarcinoma* or cancer* or carcinoma* or metasta* or neoplasm* or tumo?r).ti,ab.
21	or/17-20
22	exp Practice Guidelines as Topic/
23	practice guideline?.tw.
24	(guideline? adj2 (introduc$ or issu$ or impact or effect? or disseminat$ or distribut$)).tw.
25	clinical guideline?.tw.
26	guidance.tw.
27	recommendation.tw.
28	expert opinion.tw.
29	consensus statement?.tw.
30	evidence appraisal.tw.
31	Expert Testimony/
32	or/22-31
33	academic detailing.tw.
34	((introduc$ or impact or effect? or implement$ or computer$ or compli$) adj2 protocol?).tw.

35	((introduc$ or impact or effect? or implement$ or computer$ or compli$) adj2 algorithm?).tw.
36	clinical pathway?.tw.
37	critical pathway?.tw.
38	Patient Education as Topic/
39	Education, Medical/
40	Clinical Audit/
41	advance directive?.tw.
42	Advance Directives/
43	Fee-for-Service Plans/
44	"Peer Review"/
45	exp education,continuing/
46	(education$ adj2 (program$ or intervention? or meeting? or session? or strateg$ or workshop? or visit?)).tw.
47	(behavio?r$ adj2 intervention?).tw.
48	pamphlets/
49	(leaflet? or booklet? or poster or posters).tw.
50	((written or printed or oral) adj information).tw.
51	(information$ adj2 campaign).tw.
52	(education$ adj1 (method? or material?)).tw.
53	outreach.tw.
54	(opinion adj1 leader?).tw.
55	facilitator?.tw.
56	group detailing.tw.
57	consensus conference?.tw.
58	((effect? or impact or evaluat$ or introduc$ or compar$) adj2 training program$).tw.
59	reminder systems/
60	reminder?.tw.
61	(recall adj2 system$).tw.
62	(prompter? or prompting).tw.
63	algorithm?.tw.
64	(feedback/ OR feedback.tw.) NOT ((feedback adj1 (loop? or control? or regula$ or mechanism? or inhib$ or system? or circuit? or sensory or visual or audio$ or auditory)).tw.)
65	(57 or 58) not 59
66	chart review$.tw.
67	((effect? or impact or records or chart?) adj2 audit).tw.
68	patient education/
69	counsel$.tw.
70	compliance.tw.
71	marketing.tw.
72	exp reimbursement mechanisms/

73 fee for service.tw.
74 capitation fee/
75 "deductibles and coinsurance"/
76 cost shar$.tw.
77 (copayment? or co payment?).tw.
78 (prepay$ or prepaid or prospective payment?).tw.
79 hospital charges/
80 formular$.tw.
81 fundhold$.tw.
82 medical records/
83 medical records systems, computerized/
84 (information adj2 (management or system?)).tw.
85 peer review/
86 utilization review/
87 Mass Media/
88 Motivation/
89 incentiv$.tw.
90 allowance?.tw.
91 accreditation?.tw.
92 Licensure/
93 or/33-93
94 16 and 21 and 32 and 93

Note. Lines 1-15 contain terms relating to eligible study design; lines 17-20 contain terms relating to cancer; lines 22-31 contain terms relating to guidelines; and lines 33-93 contain terms relating to guideline dissemination and implementation interventions.
